# Survivorship care plans and information for rural cancer survivors

**DOI:** 10.1007/s11764-022-01204-0

**Published:** 2022-03-25

**Authors:** Arlen Rowe, Fiona Crawford-Williams, Belinda. C Goodwin, Larry Myers, Anna Stiller, Jeff Dunn, Joanne. F Aitken, Sonja March

**Affiliations:** 1 School of Psychology and Wellbeing, University of Southern Queensland, Ipswich, QLD Australia; 2 Centre for Health Research, University of Southern Queensland, Springfield, QLD Australia; 3 College of Nursing and Health Sciences, Flinders University, Adelaide, SA Australia; 4 Cancer Council Queensland, Fortitude Valley, QLD Australia; 5 Centre for Healthcare Transformation, Queensland University of Technology, Brisbane, QLD Australia; 6 Menzies Health Institute Queensland, Griffith University, Mt Gravatt, QLD Australia; 7 Prostate Cancer Foundation of Australia, St Leonards, Sydney, NSW Australia; 8 School of Public Health and Social Work, Queensland University of Technology, Kelvin Grove, QLD Australia; 9 School of Public Health, The University of Queensland, Springfield, QLD 4300 Australia

**Keywords:** Cancer, Survivorship, Survivorship care plan, Information, Rural, Regional and remote

## Abstract

**Purpose:**

The purpose of the study was to investigate the amount and type of survivorship care information received by cancer survivors living in rural Australia and whether this varies according to demographic factors or cancer type.

**Methods:**

Self-reported receipt of a survivorship care plan (SCP) and information on various aspects of survivorship care (e.g., managing side effects, healthy lifestyles, psychosocial advice and monitoring for recurrence) were collected from 215 cancer survivors who had returned home to a rural area in Queensland Australia after receiving cancer treatment in a major city within the previous 5 years (72% in the previous 12 months). Logistic regression was used to assess for differences across demographic factors and cancer type.

**Results:**

Only 35% of participants reported receiving a SCP and proportions of those reporting the receipt of specific information varied from 74% for information on short-term side effects to less than 30% for information on finances, chemoprevention and monitoring for signs of recurrence. No significant differences were found in the receipt of survivorship care information across demographic factors or cancer type.

**Conclusions:**

Findings suggest that cancer survivors living in rural areas are not consistently provided with adequate survivorship care information, particularly that pertaining to long-term health and recovery.

**Implications for Cancer Survivors:**

Without improved systems for delivering survivorship care information to patients returning home to rural communities after treatment, these cancer survivors risk missing out on necessary information and advice to maintain their health, wellbeing and long-term recovery.

**Supplementary Information:**

The online version contains supplementary material available at 10.1007/s11764-022-01204-0.

## Introduction

Quality survivorship care is key to promoting the health and wellbeing of cancer survivors who have completed active treatment or are no longer regularly seeing a cancer specialist. According to the of Cancer Survivorship Care Quality Framework, key domains of survivorship care include health promotion, healthcare delivery, physical effects, psychosocial effects and recurrences and new cancers (1). To facilitate quality survivorship care, information and guidance on managing long-term side effects, signs of recurrence, mental health, follow-up appointments and healthy lifestyles must be communicated to cancer survivors (2,3). Around the world rural cancer survivors (i.e. those who live outside of major cites) face unique challenges compared to their urban counterparts. With limited local access to services, many rural people living with cancer face poorer access to primary health care, financial hardship and a lack of psychosocial support (4–8). After returning home from treatment, most often conducted in metropolitan areas, the onus is typically on the individual to coordinate ongoing care, seek psychological and social support, monitor signs and symptoms and engage in health-promoting behaviour (9). Providing appropriate survivorship care information can ensure a smooth continuation of care following treatment in metropolitan areas and facilitate optimal cancer recovery and long-term wellbeing (10). In Australia specifically, 30% of cancer survivors live outside metropolitan areas and that population is growing. There is an urgent need to meet the complex, ongoing health needs of rural cancer survivors and to address geographical disparities in cancer outcomes (11).

Cancer Australia and the Institute of Medicine recommend that a survivorship care plan (SCP) is provided upon completion of treatment (2,3). A SCP is a written (or digital) record of a cancer survivor’s diagnosis, treatment, and recommended follow-up care, along with advice on maintaining their health and well-being and monitoring for recurrence. It also includes information about availability of psychological and social support (12,13). It is internationally recommended that the SCP is prepared by the treating specialist and provided to the individual and their local primary care provider. SCPs are particularly vital for rural cancer survivors returning home from treatment in major city centres where access to health and support services is limited (14).

Recent findings highlight potential inconsistencies and disparities in the delivery of vital information to cancer patients and survivors. For example, a study of over 7000 cancer patients in the USA showed that the probability of receiving a SCP was significantly lower for cancer survivors who were without a spouse, older, uninsured or with lower education (15). In a group of rural Australian cancer survivors, many still undergoing treatment, assessment and care plans (usually delivered upon diagnosis) were received by less than 40% (16). Furthermore, it was found that the receipt of written information about treatment and side effects varied according to patient characteristics such as education level and cancer type (16). However, little is known about who is receiving cancer survivorship care information in rural settings post-treatment.

To improve systems for providing survivorship care information to rural cancer survivors, it is important to understand the characteristics of those who are receiving the information currently. The current study investigates the amount and type of survivorship care information received by rural people living with cancer in Queensland Australia and whether this varies according to demographic factors or cancer type. We aim to highlight where the provision of information essential to supporting long-term health management and recovery of rural cancer survivors may need to be improved.

## Method

### Participants and procedure

The sample comprised a subset of participants in a longitudinal research project examining the experiences of rural people travelling for cancer treatment and their caregivers. Recruitment has been described in detail previously (17). This study utilised quantitative data that was collected via longitudinal questionnaires along with data from the Queensland Cancer Register. In summary, participants were guests who stayed at subsidised accommodation lodges provided by a not-for-profit cancer support organisation in Brisbane, Townsville, Toowoomba, Rockhampton and/or Cairns, while receiving cancer care. Eligible lodge guests (those aged ≥ 18 years and able to read and understand English) were invited to participate in the longitudinal study via a welcome pack which was distributed via two methods: (1) upon arrival at the lodge for their stay or (2) mailed to their home address following their stay. After receiving the pack, they were phoned to discuss the study in more depth and asked to mail back their completed consent form and questionnaire. Consenting participants completed self-administered questionnaires at the following timepoints: baseline (at recruitment), 3 months, 12 months and annually thereafter. At baseline, participants also took part in a structured interview containing demographic and health behaviour measures. Each component took approximately 45 min to complete. Ethics approval was obtained from a recognised institutional Human Research Ethics Committee (ref. H17REA152).

At baseline, 811 cancer patients consented to participate, 564 of whom completed a questionnaire at the 3-month time point which included measures of survivorship care information receipt. The current analysis was restricted to a subgroup of 215 participants who lived outside of a major city, had been diagnosed with cancer (other than non-melanoma skin cancer) within the 5 years prior to baseline, had completed treatment or had not received any treatment for their cancer for at least 3 months and were not receiving palliative care. This subgroup was chosen so that findings would reflect the recent experiences of cancer survivors who had either relocated or had been travelling back and forth to receive treatment in a major city but had since returned home to rural areas and were no longer under regular specialist care. The sample excluded those treated for non-melanoma skin cancer, in other words, a sample of people who, according to recommendations, should have received a SCP (see Supplementary File 1).

### Measures

#### Demographics

At baseline, participants provided demographic information, including residential postcode, date of birth, gender, Aboriginal/Torres Strait Islander identification and highest educational level. Participant postcode was used to ascertain area-level socio-economic status (SES) via the Socio-Economic Indexes for Areas (SEIFA; Australian Bureau of Statistics, 2016) and level of rurality via the Accessibility and Remoteness Index of Australia (ARIA; Australian Bureau of Statistics, 2011). The location of the treatment facility was coded according to the relevant Health and Hospital Service (i.e. independent statutory bodies which are responsible for delivering public health services in their areas) (18). These were then coded as metropolitan or non-metropolitan.

#### Cancer type

Most recently diagnosed primary cancer site and date of diagnosis was obtained via self-report and verified against the population-based Queensland Cancer Register (QCR). Self-report data were relied upon where diagnosis could not be verified by the QCR (*n* = 4), for example, if the patient’s diagnosis had not yet been notified to the QCR.

#### Cancer survivorship care plans and survivorship information

Receipt of a SCP was assessed via the single item “When you left the facility where you were treated did you receive a written (or digital) survivorship care plan from the medical staff” (“Yes/No”). Additionally, participants were provided with a checklist of 18 survivorship care activities, as recommended by the Australian Cancer Survivorship Centre (Wiley et al., 2015), and asked to indicate (“Yes/No”) whether they had received information relating to these items from medical staff. The items on this checklist are provided in Supplementary File 2. These items were grouped according to the domains of Cancer Survivorship Care Quality Framework including health promotion, healthcare delivery, physical effects, psychosocial effects and recurrences and new cancers (1).

### Data analysis

An a priori power analysis using G-Power 3.1 showed that a minimum sample size of *n* = 174 would be required to detect a moderate effect (*ρ* = 0.3, 1‐β = 0.80, *α* = 0.05). All data analyses and data management were conducted using the *R* statistical programme and the “dplyr” package (19–21). The proportion, and 95% CI, of people indicating “Yes” to the single SCP item and survivorship information checklist items were calculated with the “DescTools” package (22) and plotted with the “ggplot2” package (23). Logistic regression was used to assess if the receipt of SCP differed across demographic or clinical factors. The percentage of participants who reported receiving items within each of the Cancer Survivorship Care Quality domains were calculated, and logistic regression was used to assess for differences across demographic factors and cancer type. As information on ongoing adjuvant (secondary) therapy or genetic counselling is not relevant to all cancer types, these items were not included in the calculations. Alpha levels were adjusted using the Bonferroni method to correct for familywise errors.

## Results

### Sample characteristics

Participants (*n* = 215) were between 39 and 89 years of age at baseline (*M* = 64.69, *SD* = 9.53) and slightly more than half of the sample was male (52.6%). Most participants were born in Australia (77.9%), with the remainder born in the UK (10.6%), New Zealand (5.3%), and other countries (6.3%); and 72.3% of participants had received their diagnosis within the previous 12 months (see Table 1).

**Table 1:** Sample characteristics and receipt of SCPs *(N* = *215)*

	Sample		% Received SCP		
	*n*	(%)	*n*	(%)	*p*
Total	201	-	70	(34.80)	
Gender					.528
Male	113	(52.56)	38	(36.89)	
Female	102	(47.44)	32	(32.65)	
Age					.012
Less than 65 years	100	(46.51)	24	(25.81)	
65 years and over	115	(53.49)	46	(42.59)	
SEIFA					.575
Low (50th percentile or <)	173	(80.47)	56	(33.94)	
High (> 50th percentile)	42	(19.53)	14	(38.89)	
Education					.047
Year 12 or below	119	(58.05)	45	(40.91)	
Tertiary	86	(41.95)	22	(27.16)	
Remoteness					.718
Inner regional	112	(52.09)	35	(33.65)	
Outer regional/remote	103	(47.91)	35	(36.08)	
Cancer type					.850
Breast	40	(18.69)	12	(30.77)	
Head and neck	62	(28.97)	21	(36.21)	
Prostate	36	(16.82)	12	(40.00)	
Others	76	(35.51)	24	(32.88)	
Private health insurance					.067
Yes	31	(15.66)	6	(21.43)	
No or partially covered	167	(84.34)	61	(38.85)	
HHS region					.284
Metropolitan	133	(63.64)	47	(37.01)	
Non-metropolitan	76	(36.36)	20	(29.41)	

*HHS* health and hospital service, *SCP* survivorship care plan; *p* values relate to Chi square statistics testing for differences across levels of demographic variables for the number of people who reported receiving an SCP

**Fig. 1:**
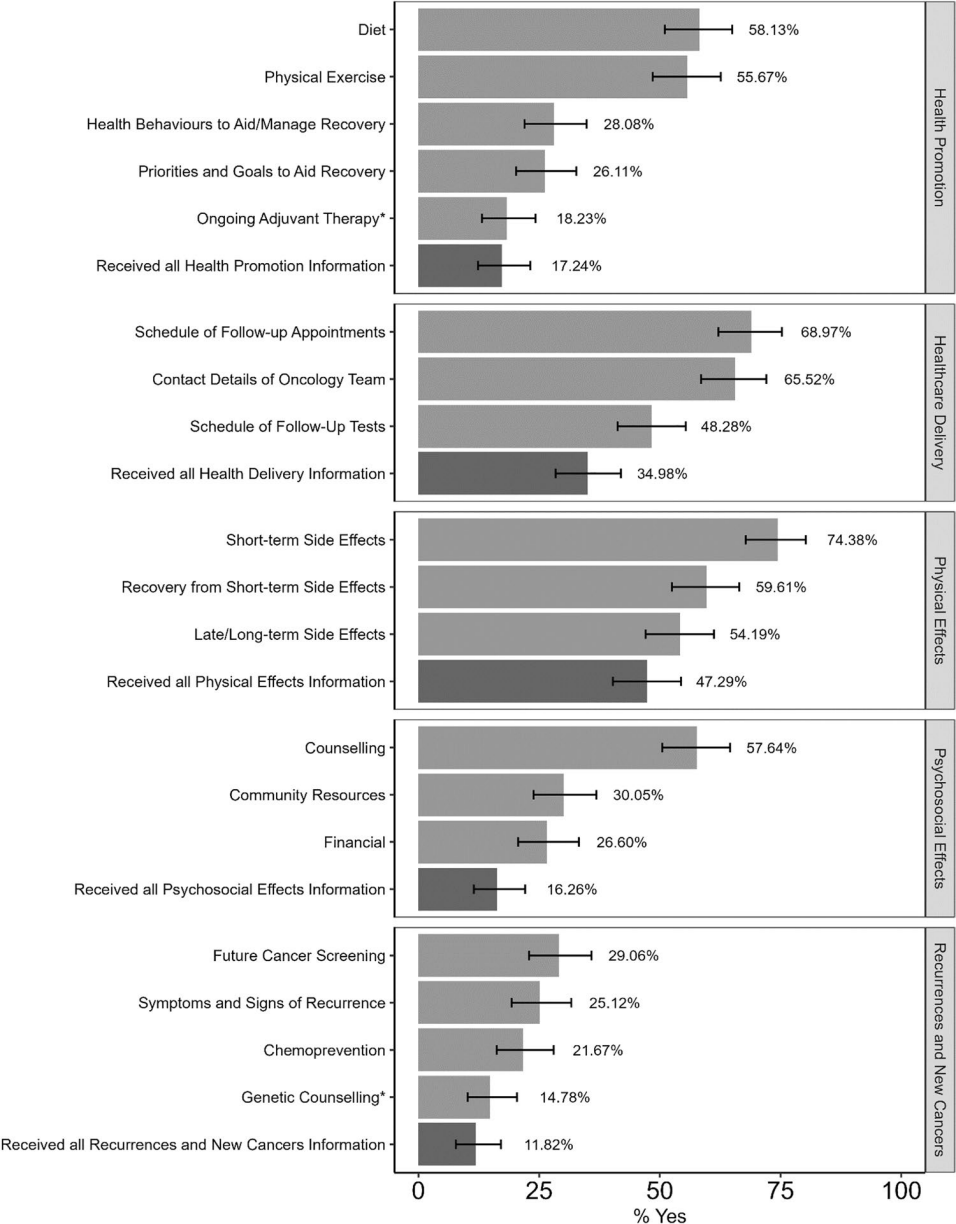
Receipt of different types of survivorship care information larger font on both vertical axis-ie both sides difficult to read

Of the 201 participants who answered the single SCP item, 34.8% (*n* = 70) reported that they had received a SCP after treatment (see Table 1). There were significantly higher odds of reporting SCP receipt for those 65 years and older when compared to those under the age of 65, OR = 2.13, 95% CI [1.18, 3.93]. The odds of reporting SCP receipt were also significantly higher for those with a year 12 level education or lower when compared to those who undertook tertiary education, OR = 1.86, 95% CI [1.01, 3.49]. However, after applying a Bonferroni adjustment for multiple test (i.e. adjusted *p* value criterion = 0.0065), there were no significant differences across demographic groups or cancer types. Figure 1 shows the percentage of people that reported receiving each item of information across grouped according to Cancer Survivorship Care Quality Framework (1). Participants were most likely to report receiving information on short-term side effects of treatment, 74.38%, 95% CI [67.80, 80.24], schedule of follow-up appointments, 68.97%, 95% CI [62.11, 75.26] and contact details of their treating oncologist/oncology team, 65.52%, 95% CI [58.54, 72.03]. With the exception of receiving information regarding genetic counselling, 14.78%, 95% CI [10.20, 20.42] and adjuvant therapy, 18.23, 95% CI [13.17, 24.24] (information not applicable to all cancer types), participants were least likely to report receiving information regarding chemoprevention, 21.67%, 95% CI [16.21, 27.98]; symptoms and signs of recurrence, 25.12%, 95% CI [19.31, 31.67]; and priorities and goals to aid recovery, 26.11%, 95% CI [20.21, 32.72].

When individual items were grouped according to the domains of Cancer Survivorship Care Quality Framework, participants were most likely to report receiving all information relating to physical effects, 47.29%, 95% CI [40.26, 54.40], and least likely to report receiving all information relating to recurrences and new cancers, 11.82%, 95% CI [7.72, 17.08]. Participants were significantly more likely to report receiving all healthcare delivery information if they were male, OR = 1.90, 95% CI [1.06, 3.45], had a year 12 or lower level of education, OR = 2.10, 95% CI [1.14, 3.97], or lived in inner regional areas, OR = 1.84, 95% CI [1.03, 3.34]. Participants were also significantly more likely to report receiving all information about physical effects if they were male, OR = 1.89, 95% CI [1.08, 3.32], or had a year 12 or lower level of education, OR = 1.94, 95% CI [1.09, 3.48]. Finally, those who have a year 12 or lower level of education were significantly more likely to report receiving all information about recurrences and new cancers, OR = 2.95, 95% CI [1.12, 9.26]. However, after correcting the *p* value criterion for multiple tests (i.e. adjusted *p* value criterion = 0.00125), there were no significant differences in the proportion of participants who reported receiving all information for each domain across demographic and clinical groups (see Table 2).

**Table 2:** Receipt of survivorship care information

				% Receiving all SCP Information									
		Effective sample		Health promotion		Healthcare delivery		Physical effects		Psychosocial		Recurrence and new cancers	
		n	(%)	%	*p*	%	*p*	%	*p*	%	*p*	%	*p*
Gender					.888		.032		.025		.832		.738
	Male	108	(53.2)	17.6		41.7		54.6		15.7		11.1	
	Female	95	(46.8)	16.8		27.4		39.0		16.8		12.6	
Age					.146		.519		.119		.453		.204
	Less than 65 years	92	(45.3)	13.0		32.6		41.3		14.1		8.7	
	65 years and over	111	(54.7)	20.7		36.9		52.2		18.0		14.4	
SEIFA					.670		.213		.573		.875		.542
	Low	162	(79.8)	16.7		37.0		46.3		16.1		11.1	
	High	41	(20.2)	19.5		26.8		51.2		17.1		14.6	
Education					.121		.017		.024		.162		.028
	Year 12 or below	112	(57.7)	20.5		42		55.4		19.6		16.1	
	University/TAFE	82	(42.3)	12.2		25.6		39.0		12.2		6.1	
Remoteness					.635		.041		.549		.770		.270
	Inner regional	106	(52.2)	16.0		41.5		45.3		17.0		9.4	
	Outer/remote	97	(47.8)	18.6		27.8		49.5		15.5		14.4	
Cancer Type					.651		.912		.086		.250		.651
	Breast	36	(17.8)	19.4		33.3		52.8		25.0		13.9	
	Head and neck	57	(28.2)	21.1		33.3		45.6		14.0		12.3	
	Prostate	35	(17.3)		14.3		40.0		62.7		20.0		5.7
	Others	74	(36.6)	13.5		33.8		37.8		10.8		12.2	
Private Health Insurance				.597		.225		.057		.130		.947	
	No or partially covered	158	(84.5)		17.7		35.4		50.0		17.1		10.8
	Yes	29	(15.5)	13.8		24.1		31.0		6.9		10.3	
Hospital Region				.524		.652		.294		.273		.353	
	Metropolitan	126	(63.6)		15.9		36.5		43.7		13.5		9.5
	Non-metropolitan	72	(36.4)		19.4		33.3		51.4		19.4		13.9

*p* values calculated via likelihood ratio test against the null model of no predictors of SCP information receipt. The Bonferroni correct *p* value criterion for 40 test is .00125

## Discussion

It is essential that all cancer survivors are equipped with comprehensive and relevant information to guide the management of their health after leaving the regular care of a cancer specialist. Despite recommendations from peak cancer bodies to this effect, only just over a third of this sample of rural cancer survivors recalled receiving SCPs after completing treatment. This is consistent with research in the USA and Australia showing only 38.6% and 37.5% of people report receipt of SCPs and treatment assessment and care plans, respectively (15,16).

While many survivors reported receiving information relating to their immediate physical and medical concerns such as short-term physical effects, oncologist contact details and follow-up appointments, less report receiving information about more distal elements of survivorship such as long-term recovery, general health promotion and monitoring for recurrence. In fact, as little one in 10 participants reported receiving all information relating to signs of recurrence and new cancers. These figures strengthen suggestions from recent qualitative research that holistic survivorship information regarding long-term wellbeing is lacking for rural cancer survivors (24). For people living in rural areas, extra challenges also exist in terms of financial hardship (due to missed work and travel) and poorer access to support services (5,25,26). For this reason, it is particularly important that those living outside of major cities receive information about resources available in their community and accessing financial support. Concerningly, only just over a quarter the current sample reported receiving advice on where to seek help about financial concerns.

The lack of information pertaining to general health and more distal outcomes may reflect the time constraints and priorities of those delivering survivorship care information upon discharge. For example, cancer treatment specialists, despite recognising the importance of providing patients with survivorship care information, often lack the time and resources needed for addressing matters beyond immediate medical needs (27,28). Nursing roles, and dedicated telehealth systems, committed specifically to delivering cancer survivorship care and support are one potential solution to this issue. Dedicating personnel to this important transitional phase may ensure comprehensive survivorship care information is provided to more patients, potentially reducing readmissions and associated healthcare costs in the future (29). RCTs that have implemented telehealth and nurse lead survivorship care have shown some success in identifying survivors needs, increasing referral rates and increasing satisfaction of care (30,31). However, other patient outcomes, such as distress and quality of life, are often not impacted by these types of interventions, and future innovations are needed to meet survivorship needs (8,30,32).

After adjusting analyses to account for familywise error, there was no statistically significant evidence that the delivery of SCPs or specific survivorship care information differed according to demographic factors or cancer type. Nevertheless, noticeably higher proportions of male, older and high school educated survivors received information which could support an emerging trend of demographic disparities. Interestingly, one similar study from the USA showed that younger and higher educated individuals were *more* likely to receive SCPs (15). The lack of significant differences may also reflect the fact that situational or environmental factors play a bigger role in the likelihood that one will receive a SCP. No differences were apparent in the delivery of survivorship information according to our broad measure of treating facility location in the current study; however, variance in site- and clinician-specific practices, attitudes and systems may affect the likelihood that adequate survivorship information is delivered (33). Given the limited and mixed evidence regarding disparity in survivorship care information receipt, it may be too early to draw any meaningful conclusions. Regardless, the implementation of a health system-wide standardised process for delivering survivorship care information would routinely prepare survivors returning to rural areas after treatment.

### Strengths and limitations

This study was the first to estimate the proportion of Australian rural cancer survivors receiving survivorship care information after treatment in a major city. It is important to note that the study relied upon patients recognising and recalling receiving an SCP: a method that may underestimate the actual receipt of such information. While not all patients may have recognised or recalled receiving a SCP document, asking about the receipt of specific types of information within the document provided more opportunities for recall and provided more detailed data regarding the types of information received. In future, more objective measures such as a review of medical records could provide more accurate estimates. It would also be useful to obtain the perspectives of health professionals delivering SCPs to identify obstacles in delivery. In terms of comparing receipt of information across groups, a conservative approach was taken in correcting for familywise error. This meant several marginal effects were interpreted as non-significant despite moderate differences in proportions. Future research with larger samples may be required to investigate the effect of demographic characteristics and cancer type.

## Conclusion

Current findings suggest that rural cancer survivors are not all being provided with adequate survivorship care information, particularly that pertaining to long-term health and recovery. Health professionals involved in cancer care may benefit from the development of a set of accepted and easily implementable recommendations to guide the routine delivery of survivorship care information to cancer survivors transitioning from hospital to home care with special consideration for those returning home to isolated areas.

## Supplementary Information

Below is the link to the electronic supplementary material.

Supplementary file1 (DOCX 33 kb)

Supplementary file2 (DOCX 14 kb)

## Data Availability

Deidentified data will be made available upon reasonable request to the corresponding author.
