# Supplementary figures and images for: Survivorship care plans and information for rural cancer survivors
Source: J Cancer Surviv. 2022 Mar 25;17(2):441–8. doi: 10.1007/s11764-022-01204-0 (PMC10036447; doi:10.1007/s11764-022-01204-0)

**Supplementary File 1. *Participant Flow Chart***


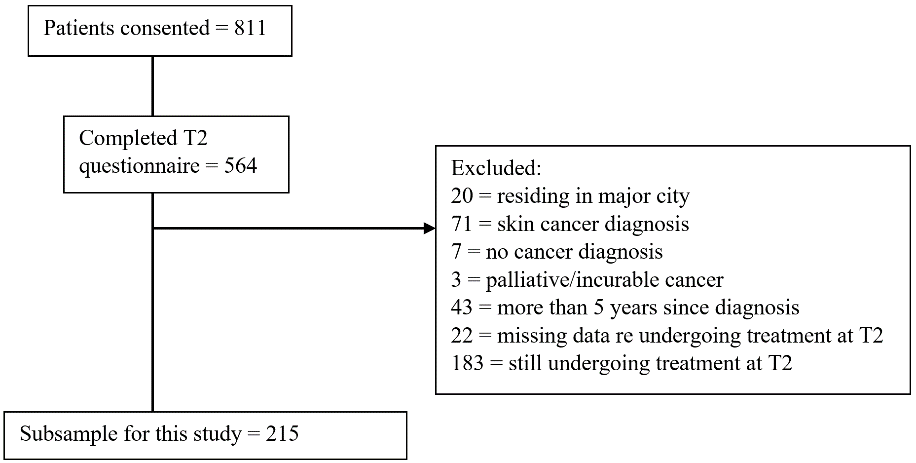

Supplement: Supplementary file 1 — Supplementary file1 (DOCX 33 kb) [file 11764_2022_1204_MOESM1_ESM.docx]
