# Supplementary material for: Survivorship care plans and information for rural cancer survivors
Source: J Cancer Surviv. 2022 Mar 25;17(2):441–8. doi: 10.1007/s11764-022-01204-0 (PMC10036447; doi:10.1007/s11764-022-01204-0)
Supplement: Supplementary file 2 — Supplementary file2 (DOCX 14 kb) [file 11764_2022_1204_MOESM2_ESM.docx]

**Supplementary File 2.**

*Survivorship Care Information*

| Domain | Item |
| --- | --- |
| Health Promotion | Information about how to manage your diet.  Information about the type and amount of physical exercise you should be doing.  Recommendations for health behaviors to aid in recovery and/or cancer management.  Advice regarding priorities and goals to aid in your recovery.  Advice regarding ongoing adjuvant (secondary) therapy. |
| Healthcare Delivery | A schedule of follow-up appointments.  The contact details for your oncologist or oncology team.  A schedule of follow-up tests that you require. |
| Physical Effects | Information about short-term side effects from your treatment.  Information about the likely course of recovery from these side effects.  Information about late or long-term side effects from your treatment. |
| Psychosocial Effects | Information about the availability of counselling.  A list of relevant resources available to you in your community.  Information or advice on where to seek help for financial concerns. |
| Recurrences and New Cancers | Instructions regarding future cancer screening for other cancers.  Details of symptoms and signs of recurrence to watch out for.  Information about chemoprevention.  Information regarding genetic counselling. |

*Note*. Grouping of items based on the Cancer Survivorship Care Quality Framework (1).
